# Supplementary material for: Dexamethasone and Dexamethasone Phosphate: Effect on DMPC Membrane Models
Source: Pharmaceutics. 2023 Mar 4;15(3):844. doi: 10.3390/pharmaceutics15030844 (PMC10053563; doi:10.3390/pharmaceutics15030844)
Supplement: Supplementary file 1 [file pharmaceutics-15-00844-s001.zip › pharmaceutics-2135352-supplementary.pdf]

SUPLEMENTARY MATERIAL

Dexamethasone and Dexamethasone phosphate: Effect on DMPC membrane models.

Candelaria Ines Cámara[1,2,*], Matías Ariel Crosio[3,4], Ana Valeria Juarez[1,2] and Natalia Wilke[3,4]

[1] Facultad de Ciencias Químicas, Departamento de Fisicoquímica, Universidad Nacional de Córdoba, X5000HUA Córdoba, Argentina;

[2] Consejo Nacional de Investigaciones Científicas y Técnicas, CONICET, Instituto de Investigaciones en Fisicoquímica de Córdoba, INFIQC, X5000HUA Córdoba, Argentina.

[3] Facultad de Ciencias Químicas, Departamento de Química Biológica Ranwel Caputto, Universidad Nacional de Córdoba, X5000HUA Córdoba, Argentina

[4] Consejo Nacional de Investigaciones Científicas y Técnicas, CONICET, Centro de Investigaciones en Química Biológica de Córdoba, CIQUIBIC, X5000HUA Córdoba, Argentina.

* Correspondence: candelaria_camara@unc.edu.ar; Tel.: +54 9 351 5353570.

Giant Unilamellar vesicles (GUVs), fluctuation analysis.

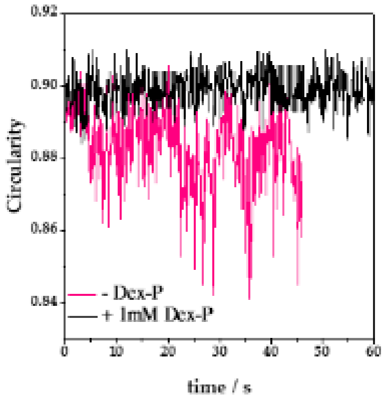

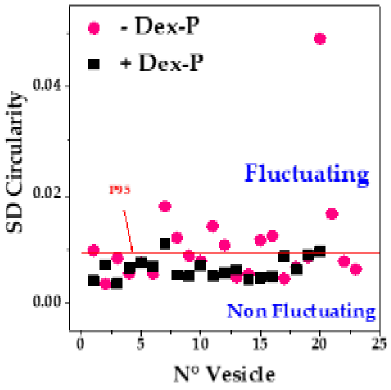

(a) **(b)**

Figure S1: (a) Demonstrative image of Circularity a function of the time for a fluctuating (pink) and non- fluctuating (black) vesicle. **(b)** Standard deviation of $\bar{C}$ (SD$\bar{C}$) as function of vesicle number. In absence (pínk) and presence (black) of Dex-P 1 mM. The straight line corresponds to the P95th value used to classify the vesicles in fluctuating or non-fluctuating.

Condition	P(95) G1	
- Dex-P	0.018	
+ Dex-P	0.0087	Less fluctuating population
-Dex	0.01041	Less fluctuating population
+ Dex	0.01276	

Table S1: Calculated P95th for each condition. The lower value of P95 correspond to the less fluctuating population and is the limit above which the vesicle is consider fluctuating.

Langmuir Isotherm:

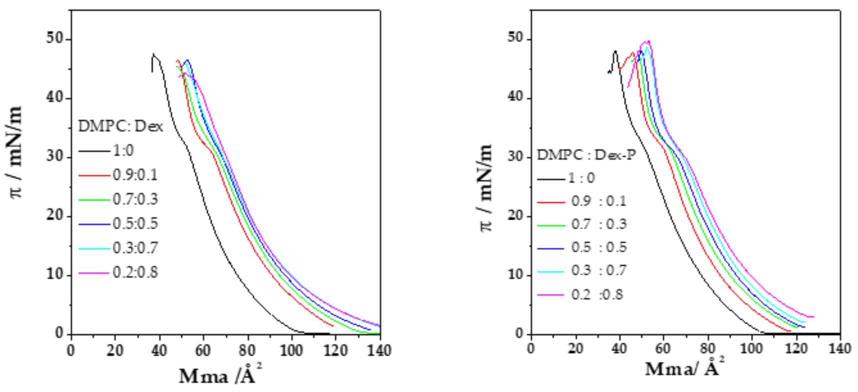

(a) **(b)**

Figure S2: Surface pressure – lipid average molecular area compression isotherm for different mixtures DMPC: X were X: **(a)** Dex and **(b)** Dex-P. Inset: Compressibility coefficient as function of the surface pressure. DMPC : X molar fraction; 1: 0 (black), 0.9:0.10(red), 0.7:0.3 (green), 0.5:0.5 (blue), 0.30:0.7 (cyan) and 0.2:0.8 (magenta). **Experimental conditions:** 150 mM NaCl, Compression rate 5 mm/min, T: 15° C.

Brewster Angle Microscopy:

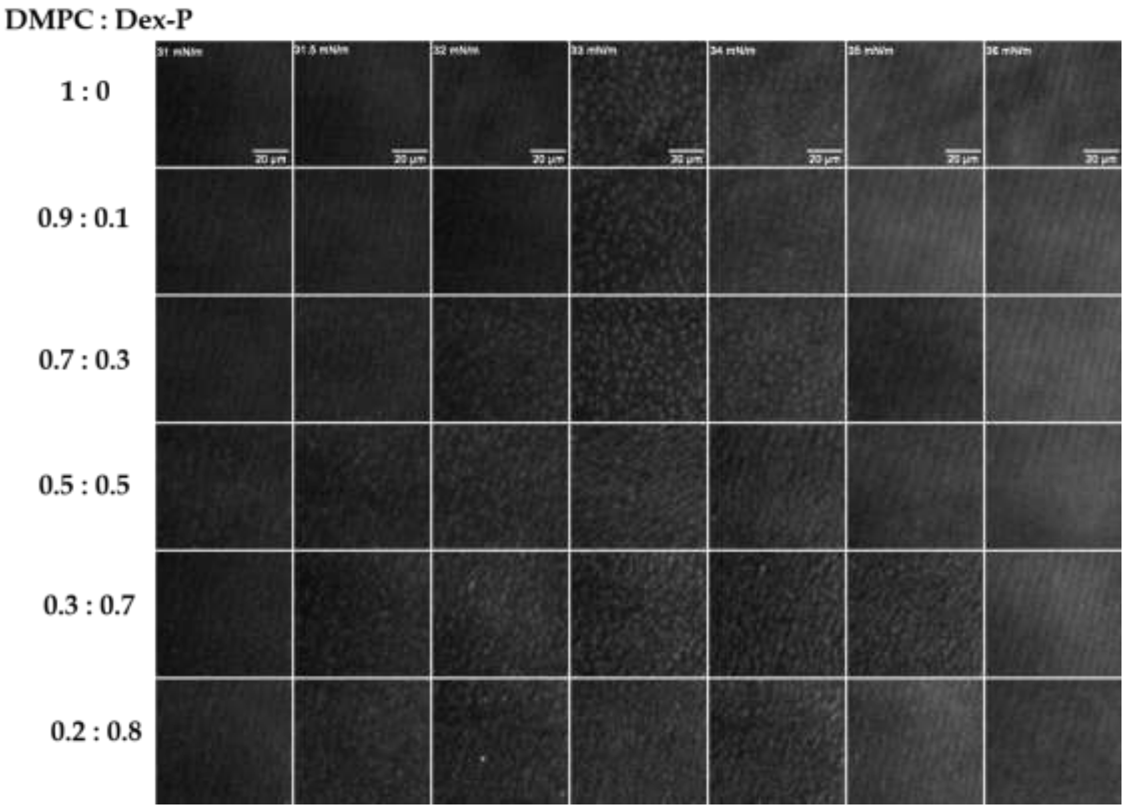

Figure S3: Brewster angle images at different surface pressures for DMP: Dex-P, the images correspond to the Langmuir isotherm shown in Figure 2b. For a better visualization the images brightness were adjusted from 0-255 to 8-60.

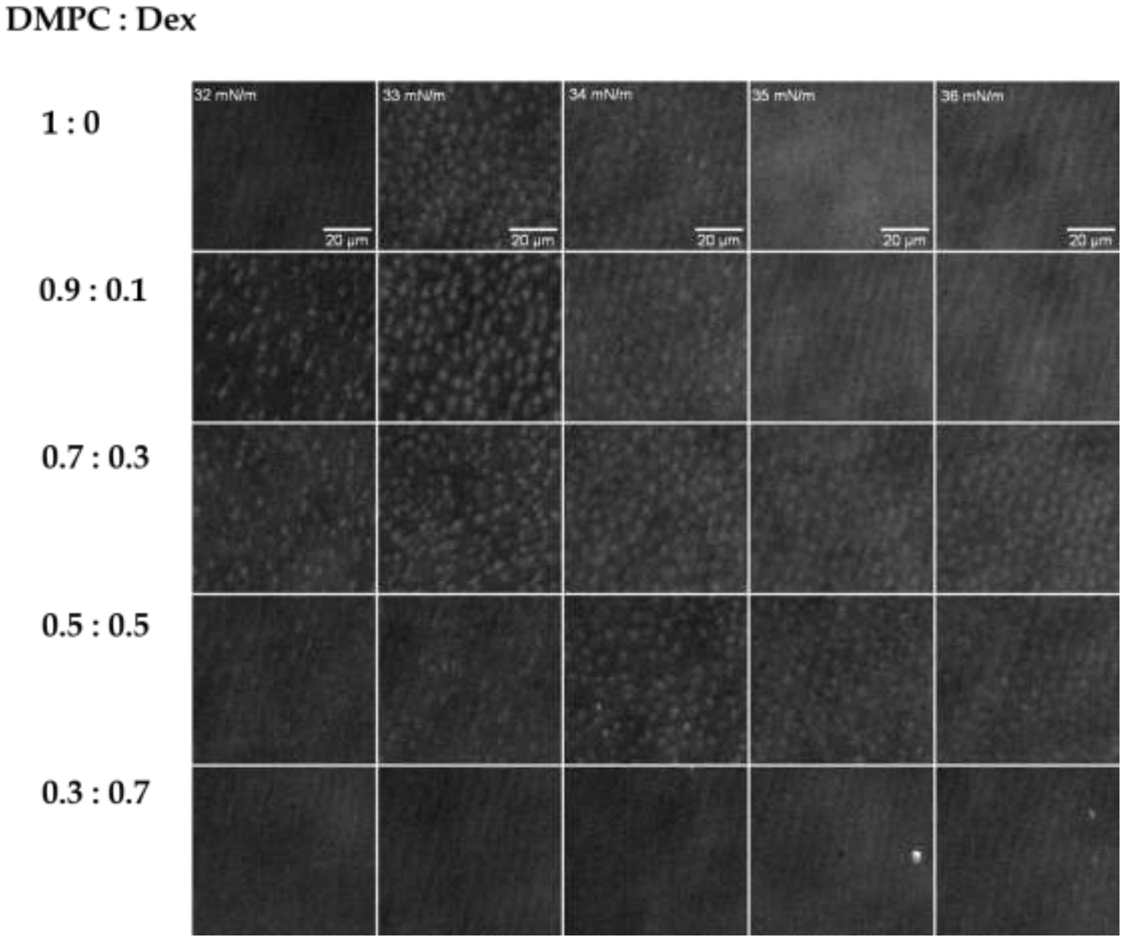

Figure S4: Brewster angle images at different surface pressures for DMPC: Dex, the images correspond to the Langmuir isotherm shown in Figure 2a. For a better visualization the images brightness were adjusted from 0-255 to 8-60.

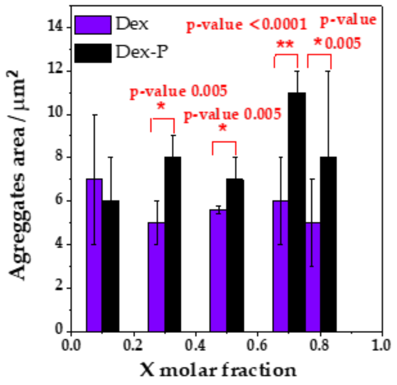

Figure S5: Aggregates area (µm^2) as function of the drug molar fraction X (X: Dex or Dex-P). The values were obtained from BAM images shown in Fig 3a-b, as an average of the different pressures (isn´t an aggregate size tendence on pressure), three images for pressure and two independent experiments at each molar fraction. An independent t-test was done at fixed molar fraction to evaluate if there is a significant difference between the aggregates area obtained for Dex and Dex-P. The significance level for the test was α = 0.05. *, ** there is a significative difference between Dex and Dex-P aggregate area.

**Dex molar fraction**	**Pearson coefficient**	**p-value**	**Dex-P molar fraction**	**Pearson coefficient**	**p-value**
0.1	-0.15	0.32	0.1	-0.22	0.22
0.3	0.19	0.21	0.3	-0.15	0.23
0.5	0.11	0.09	0.5	0.08	0.49
0.7	0.10	0.09	0.7	0.12	0.16
0.8	0.02	0.72	0.8	0.13	0.09

Table S2: Pearson coefficient and associated p-value, for the correlation analysis of aggregate reflectivity vs lateral surface pressure, at all drug molar fraction for Dex and Dex-P The significance level for the test was α = 0.05. In all cases p-value > α. There is not statical sufficient evidence to reject the non-lineal association between Rp and the lateral pressure.

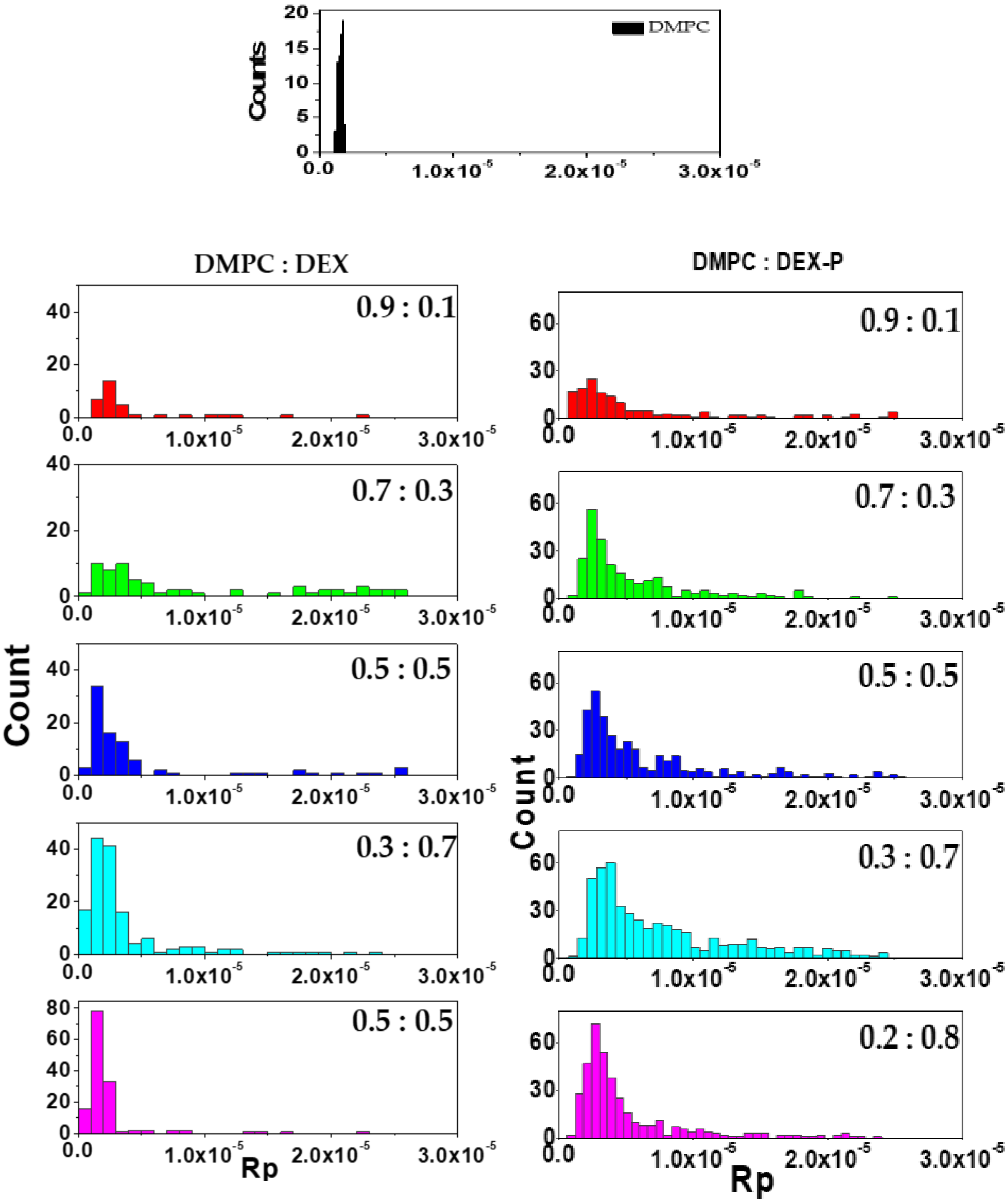

Figure S6: Absolute frequency a function of the reflectivity, for the aggregates present on BAM images. **(a)** DMPC Rp in the FC phase; DMPC:X for X: **(b)** DEX and **(c)** DEX-P. From top to down increasing X molar fraction. Bin size $0.7x10^{-7}$. The Rp value is independent of the surface pressure. The values were obtained from BAM images shown in Fig 4a-b. **Experimental conditions:** 150 mM NaCl, scan rate 5 mm/min, T: 15° C.
